# Flame Retardancy of Nylon 6 Fibers: A Review

**DOI:** 10.3390/polym15092161

**Published:** 2023-04-30

**Authors:** Xiaocheng Guo, Linjing Liu, Haisheng Feng, Dinghua Li, Zhonghua Xia, Rongjie Yang

**Affiliations:** 1 National Engineering Research Center of Flame Retardant Materials, School of Materials Science and Engineering, Beijing Institute of Technology, Beijing 100081, China; gxc17860638659@163.com (X.G.); 15901252068@163.com (L.L.); fhscppu@163.com (H.F.); zhonghua.xia@bit.edu.cn (Z.X.); yrj@bit.edu.cn (R.Y.); 2 Kingfa Sci. & Tech. Co., Ltd., Guangzhou 510663, China

**Keywords:** nylon 6, flame retardancy, fiber, surface strategy, composite

## Abstract

As synthetic fibers with superior performances, nylon 6 fibers are widely used in many fields. Due to the potential fire hazard caused by flammability, the study of the flame retardancy of nylon 6 fibers has been attracting more and more attention. The review has summarized the present research status of flame-retarded nylon 6 fibers from three aspects: intrinsic flame-retarded nylon 6, nylon 6 composites, and surface strategies of nylon 6 fibers/fabrics. The current main focus is still how to balance the application performances, flame retardancy, and production cost. Moreover, melt dripping during combustion remains a key challenge for nylon 6 fibers, and the further developing trend is to study novel flame retardants and new flame-retardancy technologies for nylon 6 fibers.

## 1. Introduction

**Scheme 1:**
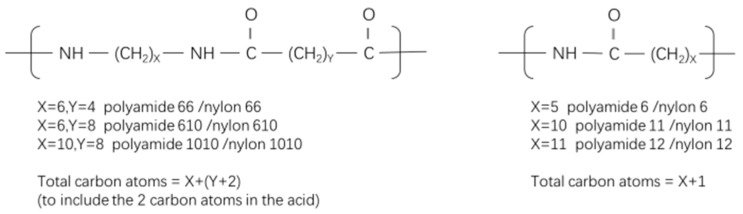
Polyamide (nylon) nomenclature.

**Figure 1:**
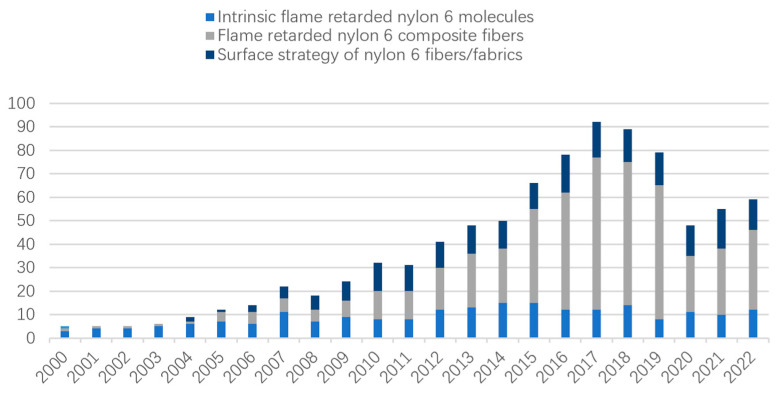
Annual trend chart of the publication number on flame retardancy of nylon 6 fibers since 2000 (data source: Web of Science).

Nylon is a synthetic thermoplastic linear polyamide (Scheme 1), including nylon 6, nylon 66, and nylon 1010. Among them, nylon 6 is one of the most widely used synthetic fibers in the world. It has excellent properties such as wear resistance, heat resistance, self-lubrication, elasticity, and toughness. Compared to other types of nylon, nylon 6 is low-cost and is widely used in fields such as textiles, electronics and appliances, automobile manufacturing, and household products. Although pure nylon 6 fibers have been widely used in the textile industry, their flammability has limited their further application. Their limiting oxygen index (LOI) value is 20% to 22%, which means that they are ignitable in the air. Furthermore, the melting droplets produced during the combustion process can lead to flame propagation, which does not meet the requirements for industrial production and application. As shown in Figure 1, the number of studies on flame retardants of nylon 6 fibers has more than doubled in recent years compared to a decade ago. It is of great significance to further enhance their flame retardancy while ensuring their mechanical properties and application performance.

In general, the combustion of polymers requires the presence of combustible materials, heat, and oxygen. A lack of any of these elements will terminate combustion. During the combustion stage, polymers undergo intense chemical changes, mainly through the rapid oxidation reaction of free radicals, releasing a large amount of heat. Heat diffuses in the air, accelerates the decomposition of polymers, promotes the mixing of free radicals and oxygen, accelerates combustion, and gradually expands the combustion area, leading to a fire. The flame-retardant mechanisms of polymers include gas-phase flame retardancy and condensed-phase flame retardancy [1]. Gas-phase flame retardancy refers to the production of free radical inhibitors by heating or combustion of materials, thereby interrupting the combustion chain reaction. Condensed flame retardancy refers to the formation of an isolation layer on the surface of a material during combustion, promoting the formation of charcoal, which acts as a shielding effect.

In the recent research, the methods to improve the flame retardancy of nylon 6 fibers include (1) intrinsic flame retardancy, that there are flame-retardant functional groups in nylon 6 molecule structure by means of reaction; (2) flame-retarded composite by physical blending, in situ polymerization, or electrostatic spinning; (3) surface strategy, including multi-layer design, fiber coating, and surface blending with other fabrics. Figure 1 illustrates that intrinsic flame-retardancy technology dominated the flame-retardant technology of nylon 6 fibers in the early 21st century. However, in the past decade, the development of flame-retardant agents has led to a significant increase in research on flame-retardant composite technology for nylon 6 fibers, as well as surface strategies for enhancing flame retardancy. 

## 2. Intrinsic Flame-Retarded Nylon 6 Molecules

Because of compatibility problems between small-molecule flame retardants and nylon 6, flame retardants often do not achieve good dispersion in the nylon 6 matrix, which directly affects the spinnability and mechanical properties of nylon 6 fibers [2]. In order to solve these problems, researchers have co-polymerized elements or structures that can exert flame-retardant effects from reactive monomers into polymer chains, thereby achieving flame-retardant effects [3].

As for the design and selection of reactive flame retardants, it is necessary to first ensure that the flame-retardant molecules are able to participate in the polymerization reaction of caprolactam to some extent, so as to achieve homogenous dispersion as much as possible. Meanwhile, flame-retardant nylon 6 materials should have sufficiently high thermal stability. This is because materials may decompose and generate flammable gases in high-temperature environments, which could cause the fire to spread. Flame-retardant materials with high thermal stability can maintain their original structure and chemical stability at high temperatures, reduce the production of flammable gases, and thus suppress the development of fires [4]. 

**Figure 2:**
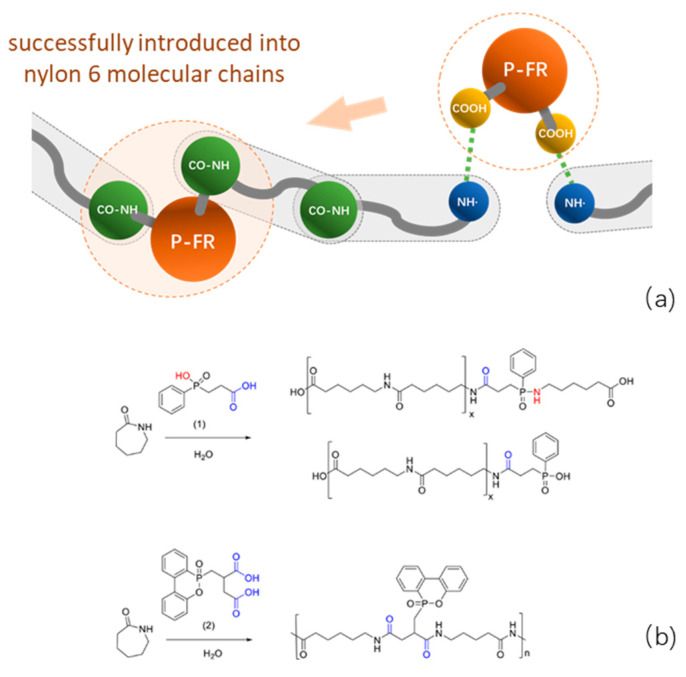
(**a**) The mechanism of how organic phosphonic acid or phosphonic acid derivatives are introduced into the nylon 6 molecules; (**b**) copolymerization reaction process of the nylon 6 backbone with some flame retardants [5].

As listed in Table 1, organic phosphonic acid or phosphonic acid derivatives are often used as reactive flame retardants of nylon 6 fibers, since they usually help achieve flame retardancy with low loading [5]. Figure 2 explains their reaction process during polymerization. It is carboxyl groups of organic phosphonic acid or phosphonic acid derivatives that participate in the copolymerization with caprolactam. Thus, the phosphorus-containing groups are introduced into nylon 6 molecules with a flame-retardancy function.

Among present flame retardants, bridged 9,10-dihydro-9-oxa-10-phosphaphenanthrene-10-oxide (DOPO) and its derivatives have been verified to be an effective intrinsic strategy of nylon 6 fibers. Apart from the reactive carboxyl groups for copolymerization, various functional groups in DOPO derivative molecules are able to enhance flame retardancy effectively. Thus, the necessary amount of flame retardants can be reduced because of the better flame-retardancy role. 

**Figure 3:**
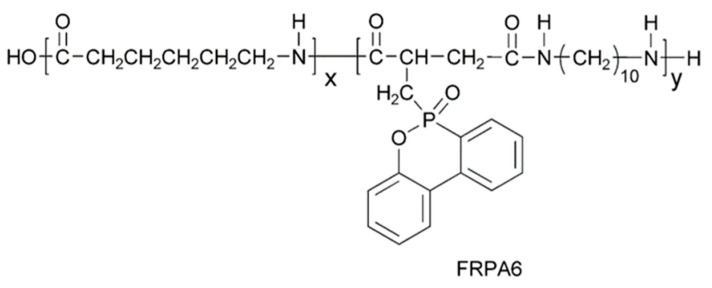
The molecular formula of FRPA6 [6].

Liu et al. [6] synthesized flame-retarded nylon 6 resin (FRPA6) by copolymerization of 9,10-dihydro-10-[2,3-di(hydroxylcarbonyl) propyl]-10-phosphaphenanthrene-10-oxide (DDP) and caprolactam (Figure 3), then prepared FRPA6 fibers by melting spinning. For copolymerization, DDP was first combined with decamethylene diamine to form a DDP salt solution, and the molecular structure was terminated with amino and carboxyl groups. Then, by polymerization with ring-opening caprolactam, DPP was successfully introduced into nylon 6 molecular chains [9]. The elements C, O, P, and N were evenly distributed in FRPA6 fibers, and no obvious structural defects were observed. With 5 wt.% DDP, the LOI value of FRPA6 fibers was raised to 28.4%.

**Figure 4:**
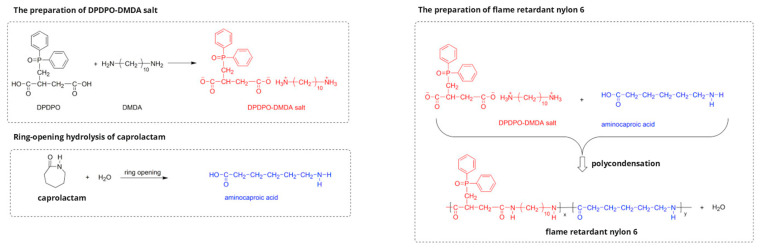
Reaction route of flame-retarded nylon 6 (PA6-DPO) [8].

Phosphine oxide derivatives are also promising flame retardants of nylon 6 fibers. By the melt polycondensation of caprolactam and 2,3-dicarboxy propyl diphenyl phosphine oxide (DPDPO), Liu et al. [8] synthesized intrinsic flame-retarded nylon 6 fibers (PA6-DPO). As shown in Figure 4, DPDPO reacted with decamethylene diamine (DMDA), while the prepolymer was prepared by the hydrolysis of caprolactam. Subsequently, the flame-retarded nylon 6 (PA6-DPO) was obtained by means of condensation polymerization.

The benzene rings of DPDPO increased the steric resistance of the molecules, so the reactivity of DPDPO’s end groups decreased and the polymerization extent was affected. The molecular weight of PA6-DPO is lower than that of pure nylon 6.

**Figure 5:**
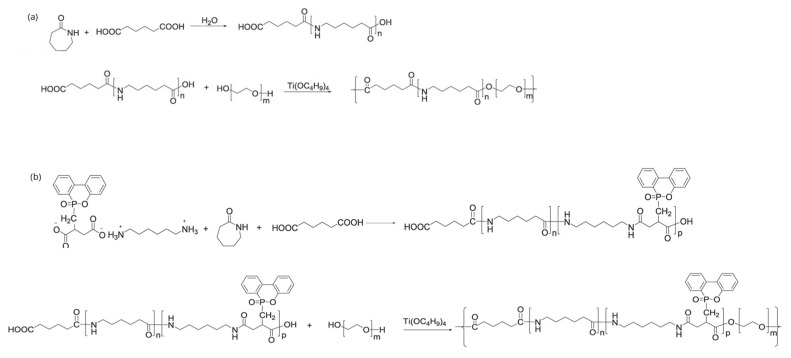
(**a**) The synthesis route of carboxyl-terminated PA6 and PA6-b-PEG; (**b**) general synthetic strategy for carboxyl-terminated PA6-xDDP and PA6PEG-xDDP blocks copolymers [10].

A “inherently” flame-retarded nylon 6 (TPAE) was synthesized by Lu et al. [10], with phosphorus-containing groups (DDP) introduced into the macromolecular chains through P-C covalent bonds, as shown in Figure 5. A TPAE molecule consists of three segments, with the polyamide (PA6) segment as the hard segment, polyethylene glycol (PEG) as the soft segment, and DDP as the flame-retardant unit [11,12]. By controlling the contents and distribution of these segments during the synthetic process, the properties of TPAEs can be regulated. The final nylon 6 products, TPAEs, achieve a UL 94 V-0 rating and an LOI value higher than 35%.

**Figure 6:**
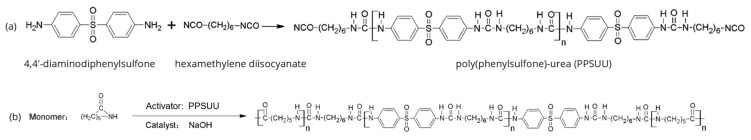
(**a**) Reaction process of poly(phenylsulfone)-urea (PPSUU) macro-activator; (**b**) reaction process of poly(phenylsulfone)-urea (PPSUU)/monomer cast (MC) nylon copolymer [13].

As shown in Figure 6, Lang et al. [13] firstly synthesized the poly(phenylsulfone)-urea (PPSUU) macro-activator by in situ anionic polymerization of 4,4’-diaminodiphenylsulfone and hexamethylene diisocyanate. Then the PPSUU segment was embedded into the nylon 6 molecular chain through copolymerization to prepare flame-retarded monomer-cast nylon 6 (MC-PA6) materials. The results show that a small amount of PPSUU can not only improve the wear resistance and impact performance of nylon 6 material but also advance the ignition time and extinguishing time and reduce the fire risk.

All the above studies [14,15,16] indicate that the average molecular weight of nylon 6 has been reduced due to the addition of DDP, DPDPO, or DDP groups. At the same time, the regularity of nylon 6 molecules is destroyed, and the crystallinity is decreased, which results in reduced fiber-breaking strength. However, the overall mechanical properties remain good.

**Figure 7:**
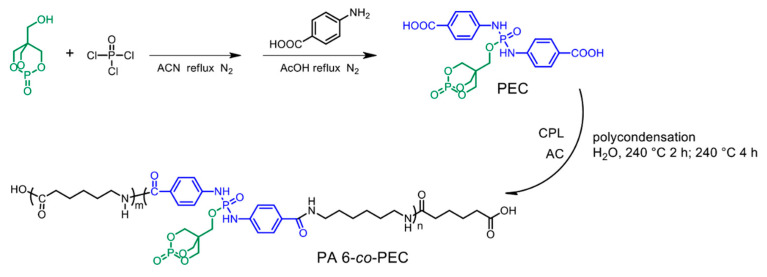
Synthesis of PEC and polymerization of PA 6-co-PEC [17].

However, Jiang et al. [17] proposed to design and synthesize a novel functional monomer (PEC) with a side-chain cage structure based on phosphorus, and prepared a thermoplastic flame-retardant polyamide polymer (PA 6-co-PEC), as shown in Figure 7. The introduction of the cage structure strengthened the entanglement of the polyamide chains [18], resulting in a 39% increase in mechanical strength and significantly improved heat resistance. In addition, due to the presence of phosphorus, gas-phase flame retardancy was achieved, leading to a significant improvement in the flame retardancy of the polyamide system. Therefore, it can be inferred that high-performance polymers with good comprehensive properties can be prepared by simultaneously adjusting the chemical composition and stereotactic configuration of the polymer.

**Figure 8:**
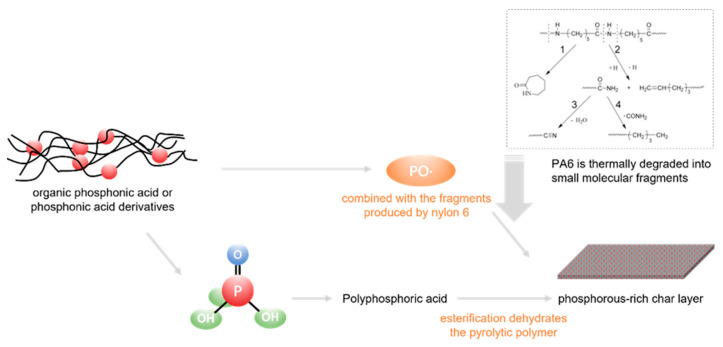
Flame-retardancy mechanisms of organic phosphorous flame retardants in nylon 6 fibers.

A detailed flame retardancy mechanism is shown in Figure 8: organic phosphoric acid or phosphoric acid derivatives produce PO· radicals and phosphoric acid during combustion. In the gas phase, the combustion reaction is hindered mainly due to the quenching effect of phosphorus-containing radicals. In the condensed phase, due to the further production of polyphosphoric acid from phosphoric acid, the phosphorus-rich char layer is eventually formed, which enhances charring behaviors and the barrier effect of the char layer. So the thermal decomposition of nylon 6 is prevented and the transfer of combustible gases, oxygen, and heat is suppressed; thereby, the flame retardancy of nylon 6 fibers is improved.

## 3. Flame-Retarded Composites

**Figure 9:**
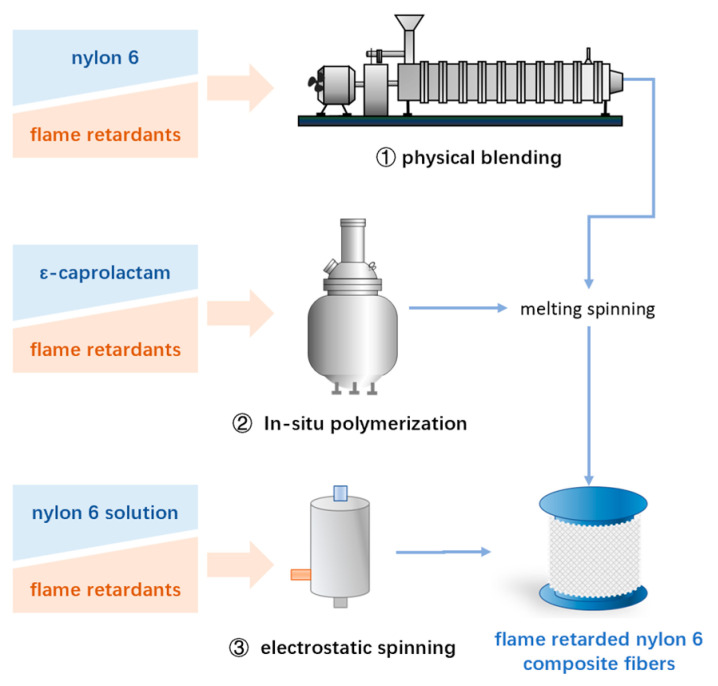
Fiber preparation process based on flame-retarded nylon 6 composites.

For the intrinsic flame retardancy of nylon 6 fibers, the necessary copolymerization of the flame retardants and caprolactam usually brings process problems and cost increases [19]. In particular, most of the inorganic flame retardants cannot be used for intrinsic technology since they cannot be introduced into nylon 6 molecules by chemical reactions. Therefore, simple and low-cost techniques such as physical blending and melting spinning have been widely used to prepare flame-retarded nylon 6 fibers [20,21]. At present, there are mainly three processing methods to prepare nylon 6 composite fibers, as shown in Figure 9.

### 3.1. Physical Blending

The better the compatibility of the flame retardants with nylon 6, the less the effect on the mechanical properties of nylon 6 composite fibers. Due to relatively good compatibility and high flame-retardancy efficiency, phosphorous-containing flame retardants are also often used in physical blending. By formulation optimization, the phosphorous-containing flame retardants can achieve a significant effect on the flame retardancy of nylon 6 composite fibers.

Di et al. [2] applied hexaphenoxycyclotriphosphazene (HPCP) for nylon 6 fibers by melting spinning method and studied the effects of HPCP on the mechanical, thermal, and combustion performances of composite fibers. HPCP mainly promotes charring through the formation of organophosphorus and aromatic products in the condensed phase. In the gas phase, HPCP behaves through the release of non-combustible gases, CO, CO_2_, and NH_3_. In addition, HPCP enhanced the fibers’ flexibility and reduced the fibers’ tensile strength. The study showed that the maximum content of HPCP for spinnability limit was 15% with 10 wt.% HPCP and the LOI value of nylon 6 composite fibers reached 28.6%. 

Zhang et al. [22] prepared multifunctional AgCl@BaSO_4_ co-precipitates with controllable nano sizes, which were melt-compounded with nylon 6 by a co-rotating twin-screw extruder. The AgCl@BaSO_4_ co-precipitate particles are well dispersed without any aggregation in the nylon 6 matrix. As a result, the stress propagates along the interfaces between nanoparticles and matrix, which is effectively conducive to the mechanical properties of the composite. The results of the UL 94 Vertical burning and LOI tests also indicate that only a small amount of AgCl@BaSO_4_ co-precipitates can obviously improve the flame retardancy of nylon 6 composites (Table 2). On this basis, the low loading ensures that the nylon 6 composites still maintain good spinnability. Furthermore, it is noteworthy that these composites have comparable antibacterial effects against Gram-positive and Gram-negative bacteria because of Ag^+^ released from AgCl.

As for the incompatible problems between flame retardants and nylon 6, the flame retardants are usually combined to improve their dispersion in the nylon 6 composites and reduce their negative influence on the mechanical behaviors by means of the interactions among the flame retardants. At the same time, higher flame-retardancy efficiency can further lower the loading of flame retardants, thus the effect on the mechanical performances can be lessened. In order to improve compatibility, more organic–inorganic hybrid agents have been designed to act as flame retardants.

Xiang et al. [23] prepared flame-retarded nylon 6 fibers by melting blending with α-zirconium phosphate (α-ZrP) and ammonium sulfamate (AS). As an inorganic agent, AS showed poor compatibility with nylon 6 and bad dispersion in the composites, which lead to a decrease in tensile strength and elongation at break. When AS is added alone by more than 5.0 wt.%, the mechanical properties of PA6/AS deteriorated significantly with brittle fracture [24]. Although the intercalation or partial exfoliation of α-ZrP is beneficial to enhance the tensile strength of the composites, high loading also can lead to particle aggregation. However, the combination of AS and α-ZrP showed a positive impact on the mechanical performances of nylon 6 composites. Compared with pure nylon 6, when the AS and α-ZrP were added at 2 wt.% and 2 wt.% individually, the tensile strength of composite fibers decreased from 3.8 ± 0.23 cN/dtex to 1.9 ± 0.07 cN/dtex, and the elongation at break increased from 33.8 ± 3.18% to 70.4 ± 3.45%. Moreover, the ion displacement reaction occurred during the combustion process, and the sulfurization of α-ZrP promoted its dispersion in nylon 6 so that the catalytic charring effect was enhanced. The synergistic effect made the LOI value increase to 32.4% and the UL 94 V-0 rating achieved, with 2 wt.% AS and 1.5 wt.% α-ZrP in nylon 6.

Liu et al. [25] designed a synergistic flame-retardant system of pentaerythritol phosphate (PEPA) and zinc diethyl phosphate (ZDP) for nylon 6. When the additional amount of PEPA and ZDP was 10 wt.% and 5 wt.%, respectively, the nylon 6 composites were still spinnable, the LOI value reached 28%, the char residue increased by 6.56%, and the total heat release decreased by 34.5%. However, the flame retardants significantly reduce the fracture strength of the fibers bundle and damage the mechanical properties of the fibers [26] while the elongation at break is maintained.

Zheng et al. [27] synthesized an inorganic-organic composite (MCN) by incorporating magnesium oxide (MgO) with graphitic carbon nitride (g-C_3_N_4_) and then blended MCN into nylon 6 to prepare flame-retarded composites. When the addition of MCN was 20 wt.%, the UL 94 rating of the PA6/MCN reached V-0 and the LOI was up to 32.1%. Compared to g-C_3_N_4_, MCN possessed a laminate structure, more holes, and a larger specific surface area. So, MCN promoted the formation of sufficient, compact, and homogenous char layers during burning. Meanwhile, the introduction of MCN effectively improved the mechanical properties and thermal stability of PA6. The enhancement of mechanical properties involves flexural strength and tensile strength.

A novel phosphorus–nitrogen flame retardant (CN-DOPO) was also synthesized by Zheng et al. via a facile method of graphitic carbon nitride (g-C_3_N_4_) and 9,10-dihydro-9-oxa-10-phosphaphenanthrene-10-oxide (DOPO) [28]. They applied CN-DOPO to nylon 6 fibers by melting blending method and studied the effect of CN-DOPO on the mechanical, thermal, and combustion performances of the fibers. The morphology of the char layer revealed that CN-DOPO formed a highly integrated char layer surface during the combustion process. Meanwhile, CN-DOPO decomposed and the release gases can inhibit combustion reaction by diluting flammable volatiles. Accordingly, the special gas-condensed phase synergistic flame-retardancy mechanism of PA6/CN-DOPO was proposed. The results showed that the LOI value was increased from 23.4% to 29.2% and the UL 94 V-0 rating was successfully passed with the 15 wt.% loading of CN-DOPO. Additionally, the mechanical properties of the composites did not deteriorate significantly.

### 3.2. In Situ Polymerization

The high melt viscosity of nylon 6 limits the dispersion behavior of flame retardants in the polymer matrix, which further affects the mechanical properties and spinnability of the composites. In situ polymerization makes it possible to control the particle size of the additives for nanoscale and obtain uniformly dispersed nanocomposites. The performances of the nanocomposites depend on their ability to maintain their nano-dispersed structure after melt blending.

**Figure 10:**
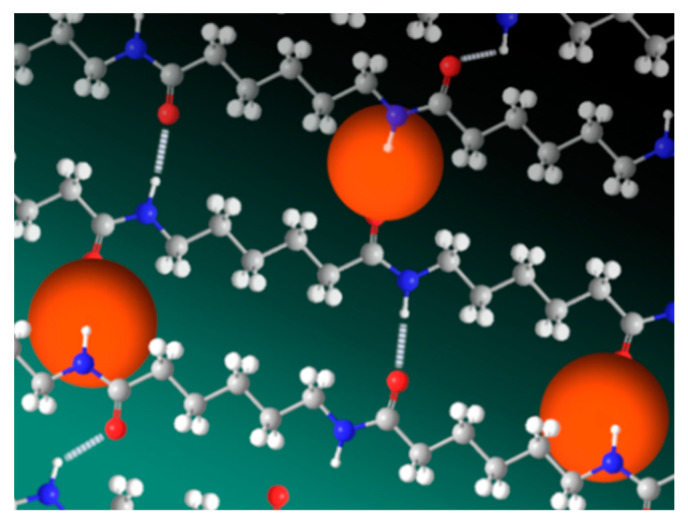
Schematic visualization of the incorporated additives that sterically hinders the interfacial adhesion of polymer chains [29].

**Figure 11:**
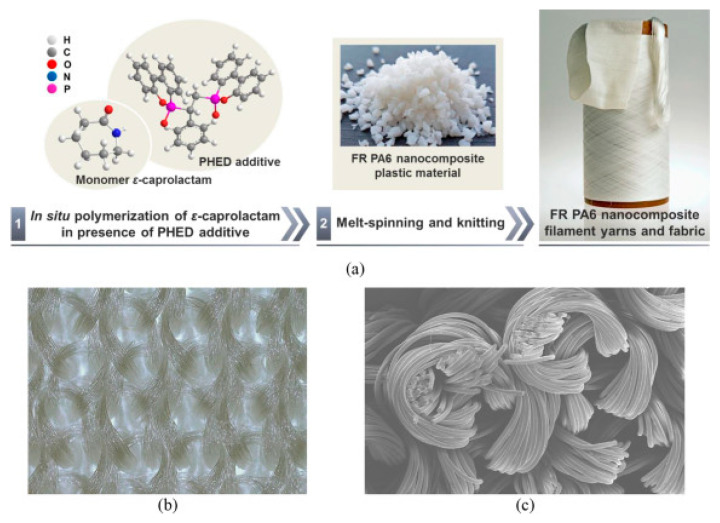
Production route for flame-retarded nylon 6 nanocomposite filament yarns and fabrics (**a**). The optical microscopy (**b**), and SEM images (**c**) of knitted fabrics corresponding to the PA6/15PHED sample [30].

Jelena et al. [29] prepared halogen-free flame-retarded nylon 6 nanocomposites by in situ water-catalyzed ring-opening polymerization of ε-caprolactam with the presence of the DOPO derivative 9,10-dihydro-9-oxa-10-phosphaphenanthrene-10-oxide (PHED). Unlike other active DOPO derivatives, PHED was physically dispersed among nylon 6 chains and formed hydrogen bonds with amide groups of nylon 6 molecules, rather than being introduced into the nylon 6 molecular structure, as shown in Figure 10. The corresponding nanocomposite filament yarns and fabrics were produced as shown in Figure 11.

The incorporated PHED reduced the internal friction and elasticity of the polymer chains. The increase in PHED content affected the crystal structure of nylon 6, which led to the lower elasticity and toughness of nylon 6 fibers. It is mainly in the gas phase that PHED enhances flame retardancy. As a DOPO derivative, it released phosphorus-containing volatiles during combustion, produced free radicals, and inhibited flame propagation. In addition, PHED lowered the thermal degradation temperature of nylon 6 and led to the earlier generation of melting droplets, which can take away the flame and heat. Thus, self-extinguishing was realized. When the PHED content was 10 wt.%, the UL 94 rating of flame-retarded nylon 6 composites reached V-0.

Melamine cyanurate (MCA) is one of the effective flame retardants for nylon 6, with the dual effects of promoting charring and intumescence. Further, the flame-retardancy efficiency can be significantly improved by means of the combination of MCA and appropriate flame retardants [31]. MCA-modified nylon 6 is mainly prepared by melt blending, and usually, the dispersion behavior is often not optimal for the spinning process.

**Figure 12:**
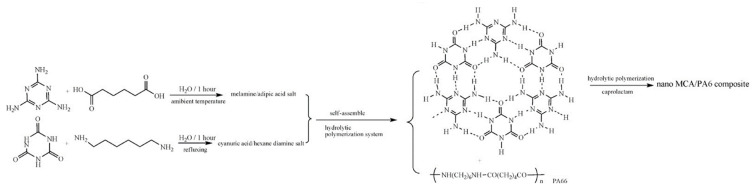
In situ synthesis mechanisms of the MCA/PA6 nanocomposites [34].

Li et al. [32] prepared melamine/nylon 6 composites by in situ polymerization, as shown in Figure 12. Melamine, cyanic acid, and MCA were difficult to dissolve in the molten state ε-caprolactam and nylon 6, so the composites prepared by direct melt copolymerization showed poor dispersion situation [33]. Here, the in situ reaction was carried out under the protection of nitrogen at 250 °C, with adipic acid-melamine (ADA-MEL) salt and cyanuric acid-hexane diamine (CA-HMDA) salt mixing with ԑ-caprolactam according to a certain ratio. The self-assembly of MEL and CA generates MCA, while the copolymerization of ADA and HMDA produces nylon 6. When the content was less than 8 wt.%, the ring-opening condensation of ԑ-caprolactam was not affected by the presence of the salt. The final products are nanoscale MCA/nylon 6 composites with no MCA groups in the nylon 6 molecular chain [34]. 

MCA with good dispersion significantly inhibited the production of melting droplets during the combustion of nylon 6 fibers, and the droplets did not ignite the skim cotton. Although the presence of MCA destroyed the crystal structure of nylon 6, resulting in a decrease in crystallinity, tensile strength, and elongation at break, the mechanical properties of MCA/nylon 6 composite fiber can still meet the requirements of industrial production and application with the 8 wt.% MCA.

### 3.3. Electrostatic Spinning

Commonly, the flame-retarded nylon 6 composites prepared by melting spinning are rarely used to prepare fibers directly because, during molten shear processing, the aggregation of flame retardants seriously affects the spinnability of the composites. Moreover, the aggregation can block the holes of the spinneret, leading to the interruption of continuous spinning.

**Figure 13:**
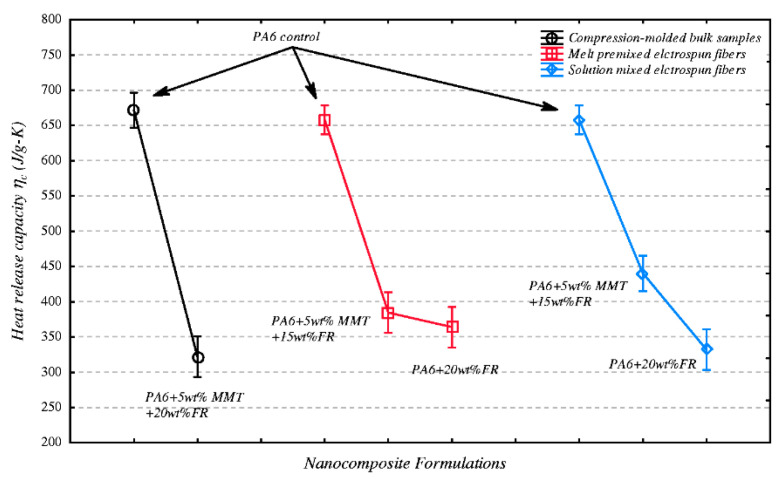
Heat release capacity of flame-retarded nylon 6 under different processing technology: compression molded bulk samples (circle point markers), melt-premixed electrospun fibers (square markers), and solution-mixed electrospun fibers (rhombus markers) [35].

Hao et al. [35] investigated the effect of electrospinning on montmorillonite clay (MMT) platelets and intumescent non-halogenated flame retardant (FR). Two methods of mixing nylon 6 with organic phosphonate flame retardants, Exolit OP1312 and MMT, were used to prepare the required spinnable fluid: (a) nylon 6 and flame retardants were extruded and mixed by high-shear melt compounding and a twin-screw extruder, and dissolved in formic acid solution; (b) polymer and additives are mixed directly in a formic acid solvent used for electrospinning. The nylon fibers prepared by high-shear melt compounding have shown better compatibility behaviors, and fewer flame-retardant particles and clay laminates were exfoliated among the fibers. Herein, MMT is more effective in the improvement of charring and the reduction of flammability by means of the premix of high-shear melt (Figure 13).

## 4. Surface Strategy

**Figure 14:**
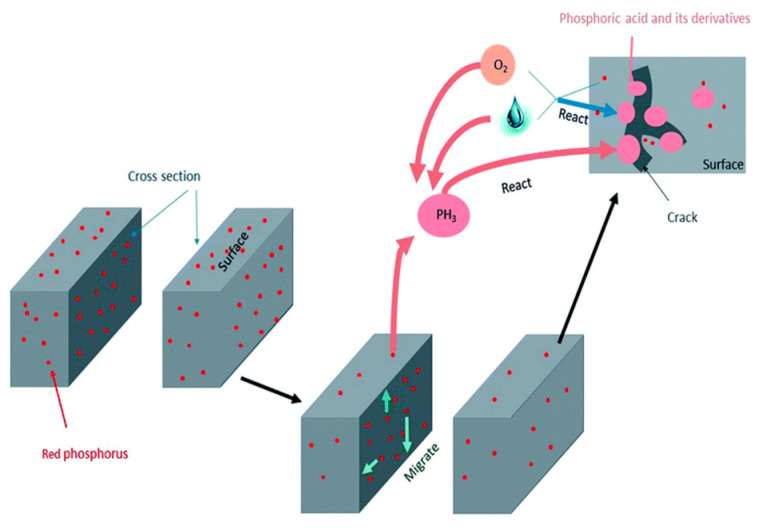
Failure behavior mechanism diagram of red phosphorus [38].

Inorganic flame retardants, such as red phosphorus, MCA, and montmorillonite, are commonly used in flame-retarded nylon 6 [36,37]. However, their poor compatibility with nylon 6 leads to not only poor dispersion during physical blending but also migration in the long-term use period, which severely affects the overall performances of the nylon 6 composite fibers [38]. In particular, as the specific surface area of nylon 6 fibers/fabrics is large, the migration of flame retardants in the fibers can cause the gradual loss of flame retardancy, as shown in Figure 14. Therefore, different surface strategies have been applied to protect the fibers/fabrics.

### 4.1. Core–Shell Structure

**Figure 15:**
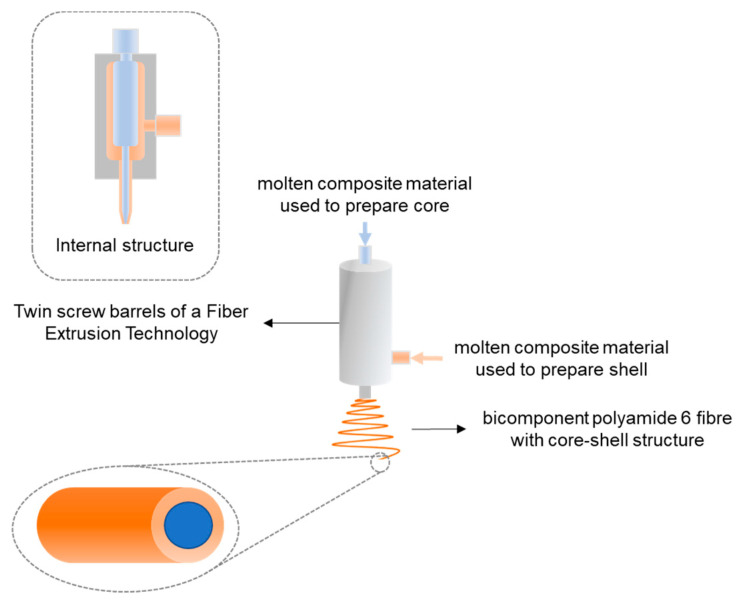
Preparation by a coaxial electrostatic spinning diagram of a two-component core–shell structure.

The core–shell structure design of flame-retarded nylon 6 fibers provides an innovative idea for low cost, excellent performance, and wide application [39]. Figure 15 shows the preparation process of nylon 6 fibers with a two-phase core–shell structure. Different devices with various structures can be designed for preparing various core–shell structures in actual production and application.

The fibers with core–shell structures function in the form of a multiphase composite structure, rather than in the form of a single-phase composite. So it is possible to avoid both the compatibility problems among flame retardants and polymer matrix and additive escaping problems during the fiber servicing period.

Horrocks et al. [40] designed bicomponent nylon 6 fibers of aluminum diethylphosphonate (AlPi) and montmorillonite nanoclay (25A) with specific locations. AlPi and 25A were separately or jointly dispersed in the core–shell structure and their effects on the mechanical properties, thermal degradation behavior, and flame-retardant properties of flame-retarded materials were studied. During combustion, a network of insulated silicates forms on the surface of the montmorillonite polymer. When AlPi is placed in the shell, the flame-retardant effect is better. In addition, when the clay flakes migrate to the fiber surface, the droplet behavior is significantly inhibited. When the amount of AlPi and clay is 10 wt.% and 2 wt.%, respectively, the flame-retardancy performance of the material is the best.

Addressing the problems associated with traditional filtering materials, Liu’s team [41] has developed an “intelligent” multifunctional material. This material can efficiently capture particulate matter (PM) below 1 μm and has a flame-retardant design. The uniqueness of this design lies in the core–shell nanofiber, with polar polymer nylon 6 as the shell and flame-retardant triphenyl phosphate (TPP) as the core. In UL 94 testing, this material achieved a self-extinguishing effect after ignition, demonstrating excellent flame retardancy. Therefore, the core–shell nanostructure has potential significance in the extension and application of multifunctional materials.

### 4.2. Surface Coating or Infiltration

Due to the large specific surface area of nylon 6 fibers/fabrics, applying inorganic or organic coatings with good water resistance is a good solution [42,43]. This can not only ensure mechanical properties but also significantly improve flame retardancy. In the industrial production process, it is also necessary to choose textile materials with bright luster and superior overall performance. Usually, surface coatings are used to improve the flame retardancy of fibers/fabrics, by promoting charring and forming stable insulation char layer.

**Figure 16:**
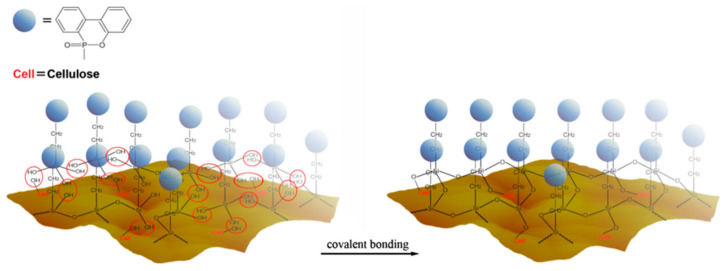
Schematic presentation of the interactions between cellulosic hydroxyl groups and DOPO-VTS [45].

Alisa Šehić [44] coated vinyl trialkoxy silane (VTS) modified with 9,10-dihydro-9-oxa-10-phosphaphenanthrene-10-oxide (DOPO-VTS) onto the surface of nylon 6 fibers using a sol–gel coating. By undergoing wetting, drying, and curing processes, the silanol groups of DOPO-VTS can form a nanostructured composite network on the surface of the fibers, resulting in improved coating durability. Figure 16 shows the synthesis mechanism. Even at a high concentration (16%), the coating does not cause substantial morphological changes on the surface of nylon 6 fibers and does not fall off during the washing process. 

The DOPO-VTS molecular structure contains both organic phosphorus-containing flame-retardant functional groups and inorganic silicon-containing groups. Therefore, its flame-retardancy mechanism involves both condensed-phase and gas-phase processes. Phosphorous-containing groups promote the early decomposition of nylon 6, generating small caprolactam molecules. Phosphorus-containing groups exhibit synergistic effects with silicon-containing groups, promoting the formation of a compact, adiabatic, and protective char layer. In comparison to another work by Aleksandra et al. [46] where DOPO derivatives were blended into nylon 6, the sol–gel coating prepared in this study exhibited a greater reduction in heat release with a lower phosphorus content (Table 3). Furthermore, the DOPO-VTS coating contributed to a reduction in the formation of molten drops of nylon 6 during burning, and the flammability of these drops was also reduced. This prevented fire spread and secondary ignition.

Liu et al. [47] prepared amine-activated cyclotriphosphazene containing siloxane (N-APESP) through a two-step synthesis. Next, they formed a Si-O-Si crosslinking structure (Si-CP) on the surface of PA6 through interfacial bonding and hydrolytic condensation of N-APESP. They proposed that the interface combined with Si-CP coating can improve the resistance to detachment, self-extinguishing properties, and water resistance of PA6 plates. The Si-CP coating played an important role in the formation of a strengthened expanded bubbly structure with high concentrations of P and Si during heating and combustion. This enhanced the barrier effect and solved the melting droplet problem.

**Figure 17:**
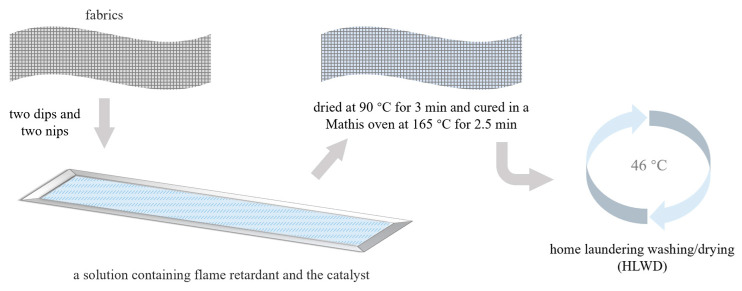
Fabrics treatment process by high-temperature bath method.

Andrzej [48] used an aqueous sodium silicate solution, known as soda water glass (WG), as a flame retardant. They prepared flame-retarded nylon 6 (PA6) fibers using the high-temperature (HT) bath method (Figure 17).

The results show that the LOI of nylon 6 fabrics varies significantly with the processing temperature. The best flame retardancy of nylon 6 fabrics is achieved when the wetting temperature is maintained at 120 °C. At this temperature, the 5% WG solution can completely permeate sodium silicate into the nylon fibers. By comparing the morphology of nylon 6 fibers after combustion, it has been found that WG can cover the residual char surface with a smooth and compact protective layer. This layer helps to reduce the volatilization of the polymer and isolate the transfer of heat. Although WG may not be as effective as other flame-retardant treatments, the high-temperature bath method has been proven to be effective in improving the flame retardancy and durability of nylon 6 fibers and fabrics [49]. Further, the flame retardancy of nylon 6 fibers and fabrics can be further improved by designing a synergistic flame-retardant system and optimizing the solution formulation [50].

### 4.3. Blended Fibers/Fabrics

Compared to melt spinning, the non-woven nylon 6 cushion can be directly prepared through electrostatic spinning [51]. It is made of sub-micron fibers with small pore sizes, high specific surface area, and volume ratios. These characteristics give the non-woven nylon 6 cushion a wide range of development value in the fields of filter media, reinforced composites, and protective clothing.

Cui et al. [52] developed a photo-grafting method to introduce carboxyl (-COOH) groups onto polyurethane-impregnated nylon-6-based ultrafine-fiber textile fabrics. A flame-retarded microfiber non-woven fabric (PNWF) was prepared by utilizing the coordination reaction between chromium ions (Cr^3+^) and -COOH. Compared with the control sample, the modified PNWF showed an increased LOI from 19.9% to 27.1%, and reduced peak heat release rate (PHRR) and peak smoke production rate (PSPR) by 32.1% and 54.2%. Furthermore, after repeated washing, the obtained PNWF still maintained 95.01% of its original LOI value, demonstrating excellent and durable flame retardancy.

**Figure 18:**
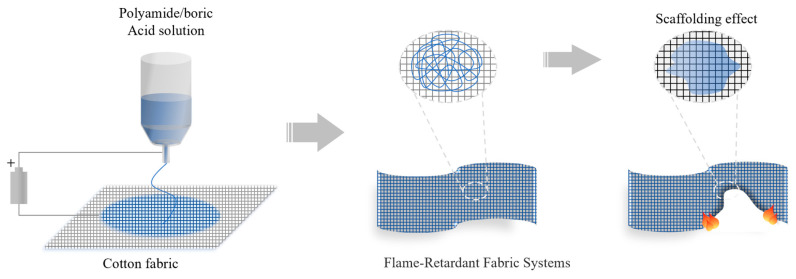
Flame-retardant fabrics systems based on electrospun polyamide/boric acid nanocomposite fibers [53]. The scaffolding effect alleviates the dripping of PA6 in the blended fabrics [54].

Selvakumar et al. [53] presented another impressive idea for modifying the flame retardancy of fabrics (Figure 18). A nylon 6/boric acid nanocomposite non-woven mat was first manufactured using electrostatic spinning technology, and then the composite fibers were coated onto cotton fabrics. During combustion, the charred cotton fibers can act as a support for the molten nylon 6, thereby reducing the formation of melting droplets from the nylon. Blending the two fibers can enhance the flame-retardant effect [54]. The introduction of boric acid nanoparticles into the coated fabrics effectively decreases the flame propagation rate. The addition of 0.5 wt.% boric acid nanocomposite fibers in the fabrics significantly alters the thermal degradation process and increases the char residue by 38%.

## 5. Conclusions and Perspectives

The addition of flame retardants often affects the mechanical or spinning behavior of nylon 6 fiber/textiles, so the main challenge in developing flame-retarded nylon 6 is to find a balance between flame-retardancy performance, processing performance, and application performance. In practical applications, flame-retardant nylon 6 material not only meets the flame-retardant requirements, but also needs to ensure that mechanical properties are not damaged and consider dielectric properties, insulation properties, processing properties, and spinnability. According to different application scenarios, thermal conductivity, thermal insulation, and antibacterial properties should also be given attention. Indeed, due to the advantages of blending additives, composite materials are currently widely used, but their impact on mechanical and spinning behavior cannot be ignored. Therefore, we hope to achieve greater achievements in intrinsic flame retardancy. The next milestone that the field is trying to achieve is to achieve intrinsic flame retardancy with excellent drip resistance, processing performance, and mechanical properties through the design of new reactive flame retardants or flame-retardant functional groups.

With the increasing awareness of environmental protection among people, phosphorus-containing flame retardants are widely used as low-toxicity flame retardants because they can produce flame-retardant effects in both gas and condensed phases. Based on recent advances, the flame-retardancy technology of nylon 6 fibers has made significant progress through the use of new reactive flame retardants, dispersion-promoting processing methods, and fiber surface strategies. By introducing phosphorous-containing organic functional groups into the nylon 6 molecules, some flame-retarded nylon 6 fibers can be prepared with low flame-retardant content, high flame-retardancy efficiency, and little impact on mechanical performances. However, the strict copolymerization conditions limit the development of intrinsic flame retardancy in nylon 6 fibers. Currently, nylon 6 composite fibers are still the most widely used, especially for inorganic flame retardants with low cost and minimal impact on the environment. Regarding processing technology, surface strategies of nylon 6 fibers/fabrics are more convenient, and innovative work in this area has been reported continuously. In conclusion, novel reactive flame retardants are expected to provide excellent overall performances for nylon 6 fibers, and new synergistic flame-retardant systems hold promise for improved flame retardancy with less negative impact. Surface strategies of fibers/fabrics also offer new concepts and perspectives for flame retardancy with convenient processing methods. 

## Figures and Tables

**Table 1:** Some phosphorous compounds and reactive flame retardants of nylon 6 fibers.

Flame Retardants	Molecular Structures	References
3-hydroxyphenylphosphinylpro-pionic acid (3-HPP)	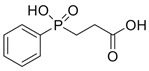	[5]
9,10-dihydro-10-[2,3-di(hydroxylcarbonyl) propyl]-10-phosphaphenanthrene-10-oxide (DDP)	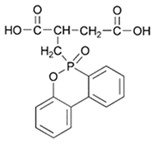	[6]
1-oxyphosa-4-hydroxymethyl-2,6,7-trioxabicyclic [2,2.2] octane (PEPA)	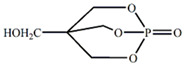	[7]
2,3-dicarboxy propyl diphenyl phosphine oxide (DPDPO)	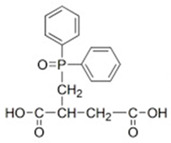	[8]

**Table 2:** Flame retardancy of the nylon 6 composites (PA6-AgCl@BaSO_4_) [22].

Sample	LOI%	Ignition of Cotton (p/p)	UL 94 Rating
Pure PA6	25.5 ± 0.2	Yes (5/5)	V-2
0.5 wt.%	26.4 ± 0.1	Yes (5/5)	V-2
1.5 wt.%	30.0 ± 0.2	No (3/5)	V-1
2.5 wt.%	30.6 ± 0.3	No (0/5)	V-0

**Table 3:** Relevant pyrolysis combustion flow calorimetry data for PA6, DiDopoMeO/PA6, DiDopoEDA/PA6, and Exolit^®^ OP 1230/PA6 [44,46].

Formulation	TM (°C)	pHRR (W·g^−1^)	THR (kJ·g^−1^)	P content (wt.%)
PA6	480 ± 3	528 ± 8	26.1 ± 0.3	–
PA(16P + 8Si)	430 ± 1	459 ± 2	23.5 ± 0.4	1.22
DiDopoMeO/PA6	422 ± 4	486 ± 12	24.4 ± 0.2	2.28 ± 3.3 × 10^−2^
DiDopoEDA/PA6	429 ± 3	566 ± 11	24.4 ± 0.2	2.03 ± 0.1 × 10^−2^
Exolit^®^ OP 1230/PA6	455 ± 1	495 ± 3	24.7 ± 0.2	4.07 ± 1.8 × 10^−2^

## Data Availability

The data presented in this study are available on request from the corresponding author.
